# Efficient expansion of human keratinocyte stem/progenitor cells carrying a transgene with lentiviral vector

**DOI:** 10.1186/scrt338

**Published:** 2013-10-18

**Authors:** Daisuke Nanba, Natsuki Matsushita, Fujio Toki, Shigeki Higashiyama

**Affiliations:** 1 Senior Research Fellow Center, Ehime University, Shitsukawa, Toon, Ehime 791-0295, Japan; 2 Division of Cell Growth and Tumor Regulation, Proteo-Science Center, Ehime University, Shitsukawa, Toon, Ehime 791-0295, Japan; 3 Translational Research Center, Ehime University Hospital, Ehime University, Shitsukawa, Toon, Ehime 791-0295, Japan; 4 Department of Biochemistry and Molecular Genetics, Graduate School of Medicine, Ehime University, Shitsukawa, Toon, Ehime 791-0295, Japan

## Abstract

**Introduction:**

The development of an appropriate procedure for lentiviral gene transduction into keratinocyte stem cells is crucial for stem cell biology and regenerative medicine for genetic disorders of the skin. However, there is little information available on the efficiency of lentiviral transduction into human keratinocyte stem/progenitor cells and the effects of gene transduction procedures on growth potential of the stem cells by systematic assessment.

**Methods:**

In this study, we explored the conditions for efficient expansion of human keratinocyte stem/progenitor cells carrying a transgene with a lentiviral vector, by using the culture of keratinocytes on a feeder layer of 3 T3 mouse fibroblasts. The gene transduction and expansion of keratinocytes carrying a transgene were analyzed by Western blotting, quantitative PCR, and flow cytometry.

**Results:**

Polybrene (hexadiamine bromide) markedly enhanced the efficiency of lentiviral gene transduction, but negatively affected the maintenance of the keratinocyte stem/progenitor cells at a concentration higher than 5 μg/ml. Rho-assiciated kinase (ROCK) inhibitor Y-27632, a small molecule which enhanced keratinocyte proliferation, significantly interfered with the lentiviral transduction into cultured human keratinocytes. However, a suitable combination of polybrene and Y-27632 effectively expanded keratinocytes carrying a transgene.

**Conclusions:**

This study provides information for effective expansion of cultured human keratinocyte stem/progenitor cells carrying a transgene. This point is particularly significant for the application of genetically modified keratinocyte stem/progenitor stem cells in regenerative medicine.

## Introduction

Recombinant retroviruses enable a transgene to permanently integrate into the genome of a target cell, and are widely used as a vector in gene therapy [1,2]. Efficient and safe gene transduction into stem cells with viral vectors is indispensable for successful regenerative medicine, including the generation of genetically modified tissue stem cells and induced pluripotent stem (iPS) cells derived from patients affected by genetic disorders [3,4]. Transduction and silencing of genes with viral vectors are also essential for analyzing stem cell function during development, homeostasis and tumorigenesis. Thus, the development of highly efficient and less-cytotoxic viral gene transduction into stem cells is crucial for advances in stem cell-based regenerative medicine and stem cell biology.

Human skin contains keratinocyte stem cells that are clonogenic when cultivated on a feeder layer of mouse 3 T3 fibroblasts, and show significant proliferative capacity in culture [5]. *Ex vivo* maintenance and expansion of human keratinocyte stem cells have achieved the autologous transplantation of confluent sheets of cultured keratinocytes onto patients with extensive burns [6]. The culture of keratinocyte stem cells has also enabled a gene therapy for a genetic disorder of the skin. De Luca and his colleagues [7] isolated epidermal keratinocyte stem cells from a patient who carries a null allele and a single point mutation in the *LAMB*3 gene encoding laminin beta 3 subunit, and thus is affected by junctional epidermolysis bullosa. They transduced the full-length *LAMB3* cDNA into the keratinocyte stem cells with a retroviral vector, and prepared genetically corrected cultured epidermal grafts. The grafts were engrafted and remained stable for at least one year in the absence of blisters, infections, inflammations or immune response. Thus, gene transduction into cultured keratinocyte stem cells assures the healing or alleviation of inherent genetic disorders of the skin.

Polybrene (hexadimethrine bromide), a cationic polymer, has been widely used to increase the efficiency of retroviral transduction [8]. Cationic polymers, including polybrene, enhance virus adsorption on the surface of the cell by neutralizing the negative electrostatic repulsion between the cell surface and the virus particles [9,10]. It has been reported that polybrene is utilized for introduction of DNA into keratinocytes as a DNA carrier [11,12], and that it also enhances gene transduction into keratinocytes with adenoviruses [13,14] and retroviruses [15,16]. Besides polybrene, van den Bogaard *et al*. have recently reported that Rho-associated kinase (ROCK) inhibitor Y-27632 enhances lentiviral transduction into human keratinocytes [17]. However, there is little information available on how polybrene and Y-27632 impact the efficiency of lentiviral transduction into human keratinocytes, keratinocyte proliferation and maintenance of the stem/progenitor cells. Here, we provide information for an efficient and less-cytotoxic gene transduction with a lentiviral vector into human epidermal keratinocyte stem/progenitor cells cultured on a feeder layer of 3 T3 mouse fibroblasts by using polybrene and Y-27632.

## Methods

### Cell culture

The culture of normal human epidermal keratinocytes (strain no. 685389; KURABO, Osaka, Japan) isolated from the foreskin of newborns was previously described [18]. Briefly, frozen keratinocytes were thawed and cultivated at clonal density on a feeder layer of mitomycin C-treated 3 T3-J2 cells at 37°C as described [19,20]. The medium was changed every four days. Cells were used between passage 4 and 10. For determination of colony-forming efficiency, 1 × 10^4^ keratinocytes were cultured as described above. Cultures were maintained for eight days, and subsequently fixed in 3.7% buffered formaldehyde, and stained with 1% rhodamine B (Sigma-Aldrich, St. Louis, MO, USA).

### Preparation of lentiviral vector

A plasmid for lentiviral expression of enhanced green fluorescent protein (EGFP) was generated by subcloning the EGFP cDNA from pEGFP-N1 (Clontech, Palo Alto, CA, USA) into the HIV-based self-inactivating lentiviral expression vector plasmid pCSII-CMV-MCS [21] (RIKEN BioResource Center, Tsukuba, Japan). The EGFP expression cassette under the control of CMV promoter was introduced upstream of woodchuck hepatitis virus posttranscriptional regulatory element in the transfer plasmid pCSII-CMV-MCS. Lentiviral vector pseudotyped with vesicular stomatitis virus glycoprotein was generated by standard DNA transfection. HEK293T cells were transfected with transfer, envelope and packaging plasmids using lipofectamine LTX (Invitrogen, Carlsbad, CA, USA). Viral vector particles were ultracentrifuged at 100,000 × g for 1 hr, and resuspended in Dulbecco-modified Eagle’s medium. To measure the functional titer, HEK293T cells were seeded into a 24-well culture plate, transduced with proper concentrations of viral vectors, and the functional titer was analyzed by flow cytometry (FACS Calibur; Becton Dickinson, Franklin Lakes, NJ, USA). The multiplicity of infection (MOI) of 1 was defined as a number of functional lentiviral particles which was sufficient to introduce a transgene into HEK293 cells with 100% efficiency.

### Lentiviral infection

**Figure 1:**
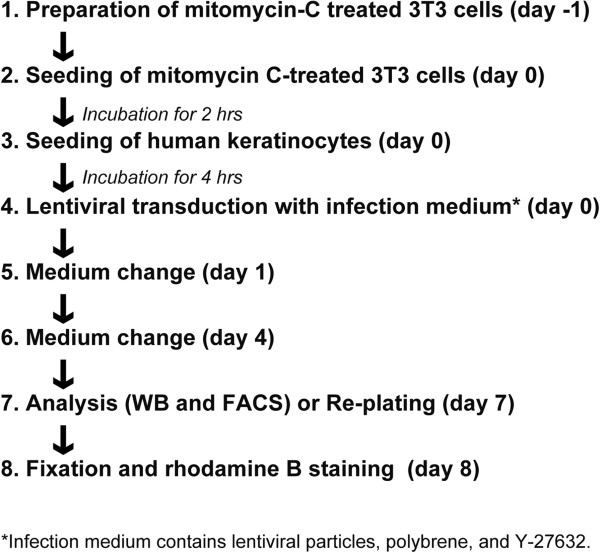
**The flowchart of lentiviral transduction and analysis of gene transduction.** The procedure of lentiviral transduction into human keratinocyte stem/progenitor cells on a feeder layer of 3 T3 mouse fibroblasts, and subsequent analysis of gene transduction. See also Methods.

A total of 10^4^ keratinocytes were seeded in a 12-well cell culture plate with mitomycin C-treated 3 T3-J2 cells. Keratinocytes were further incubated for four hours and then infected by replacing the infection medium containing lentiviral particles, polybrene and Y-27632 at various concentrations. The infection medium was removed and replaced by fresh medium after overnight incubation. The treatment with Y-27632 continued until Day 4 in culture. After seven days of cultivation, the expression level of the transgene was analyzed by Western blotting and flow cytometry. Polybrene (hexadiamine bromide) and Y-27632 were purchased from Sigma-Aldrich and Wako (Osaka, Japan), respectively. The procedure of lentiviral infection and subsequent analysis of gene transduction are described in Figure 1.

### Western blotting

A total of 10^4^ keratinocytes were seeded in a 12-well cell culture plate with mitomycin C-treated 3 T3-J2 cells, grown for seven days, and analyzed by Western blotting, as previously described [18]. The luminescence signals were detected by using ImageQuant LAS4010 (GE Healthcare, Buckinghamshire, UK). Primary antibodies used were as follows: rabbit polyclonal antibody to GFP (#598; 1:1,000; Medical & Biological Laboratories, Nagoya, Japan), and mouse monoclonal antibody to α-tubulin (#T6199; 1:5,000; Sigma-Aldrich). Secondary antibodies were horseradish peroxidase (HRP)-conjugated goat anti-mouse and rabbit IgG (#115-055-174 and #211-032-171; 1:5,000; Jackson ImmunoResearch, West Grove, PA, USA). Relative density of EGFP bands was measured with Volocity (PerkinElmer, Branchburg, NJ, USA) and normalized with density of α-tubulin bands. The values (means ± SD) were determined based on results in three independent experiments.

### Cell proliferation assay

A total of 10^4^ keratinocytes were seeded in a 12-well cell culture plate with mitomycin C-treated 3 T3-J2 cells. After seven days of cultivation, keratinocytes were trypsinized and stained with 0.4% Trypan blue (Invitrogen) to identify dead cells. Then, the number of cells was counted with a Countess™ automated cell counter (Invitrogen), which could eliminate the dead cells and large mitomycin C-treated 3 T3-J2 fibroblasts with irregular shape. The values (means ± SD) were determined based on results from three independent experiments.

### Immunofluorescence microscopy

A total of 2 × 10^3^ keratinocytes were seeded at clonal density in a 35-mm cell culture dish with mitomycin C-treated 3 T3-J2 cells, grown for six days, and analyzed by immunofluorescence microscopy, as previously described [18]. Rat monoclonal antibody to α6 integrin (clone GoH3; 1:200; Becton Dickinson), and mouse monoclonal antibody to p63 (clone 4A4; 1:500; Millipore, Bedford, MA, USA) and involucrin (clone SY5; 1:1,000; Abcam, Cambridge, UK) were used as primary antibodies. Cy3-conjugated goat anti-rat IgG and anti-mouse IgG (#112-165-167 and #715-025-140; 1:500; Jackson ImmunoResearch) were used as secondary antibodies.

### Flow cytometry

A total of 2 × 10^5^ keratinocytes were seeded in a 60-mm cell culture dish with mitomycin C-treated 3 T3-J2 cells, transduced *EGFP* cDNA with lentivirus vector in presence or absence of Y-27632, and grown for seven days. Trypsinized and resuspended keratinocytes were incubated with mouse monoclonal antibody against α6 integrin (clone 17D11; 1:20; a gift from Dr. Hirako, Nagoya University, Nagoya, Japan) for 1 hr on ice. Keratinocytes were incubated with phycoerythrin (PE)-conjugated goat polyclonal antibody against mouse IgG (sc-3738; 1:250; Santa Cruz Biotechnology, Santa Cruz, CA, USA) for 1 hr on ice. After three washes with Hank’s Balanced salt solution containing calcium and magnesium (HBSS (+)), the cells were then resuspended in HBSS (+) and analyzed by using the FACSCan flow cytometer (Becton Dickinson).

### Calculation of EGFP-positive keratinocytes and 3 T3 feeder cells

The percentages of EGFP-positive keratinocytes and 3 T3 feeder cells were calculated by the following equations: EGFP-positive keratinocytes (%) = (% of EGFP-positive and ITGA6-positive cells)/(% of EGFP-positive and ITGA6-positive cells +% of EGFP-negative and ITGA6-positive cells) × 100, and EGFP-positive 3 T3 feeder cells (%) = (% of EGFP-positive and ITGA6-negative cells)/(% of EGFP-positive and ITGA6-negative cells +% of EGFP-negative and ITGA6-negative cells) × 100. The values (means ± SD) were determined based on results from three independent experiments.

### Quantitative PCR

A total of 2 × 10^5^ keratinocytes were seeded in a 60 mm cell culture dish with mitomycin C-treated 3 T3-J2 cells, transduced *EGFP* cDNA with lentivirus vector in the presence or absence of Y-27632, and grown for seven days. Genomic DNA was extracted from keratinocytes and 3 T3-J2 cells with lysis buffer consisting of 50 mM Tris–HCl (pH 8.0), 100 mM NaCl, 20 mM EDTA, 1% SDS, and 5 units/mL of Proteinase K (Sigma-Aldrich). The DNA (50 ng) was subjected to quantitative PCR using *Power* SYBR Green PCR Master Mix Reagents and a 7300 Real-Time PCR System (Applied Biosystems, Foster City, CA, USA). Relative copy number of the transgene was calculated based on the ∆∆Ct method by normalization using the Ct-value of the human or mouse phosphoglycerate kinase (*PGK*) gene. The detection primers used were as follows: 5′-GTGAACGGATCTACAAATGGCAG and 5′- GTCTGTTGCTATTATGTCTACTA for the recombinant lentiviral vector, 5′- TGATTATTGGTGGTGGAATGGCTT and 5′-TGGAGGTCAGCATCTATACTAAGA for the human *PGK* gene, and 5′-TGCTAGACAAAGTCAATGAGATGA and 5′-TGATATGCAACCACTGTGAAAGGGT for the mouse *PGK* gene. The values (means ± SD) were determined based on results from three independent experiments.

## Results

### Polybrene enhances transduction efficiency but reduces growth potential of keratinocytes

**Figure 2:**
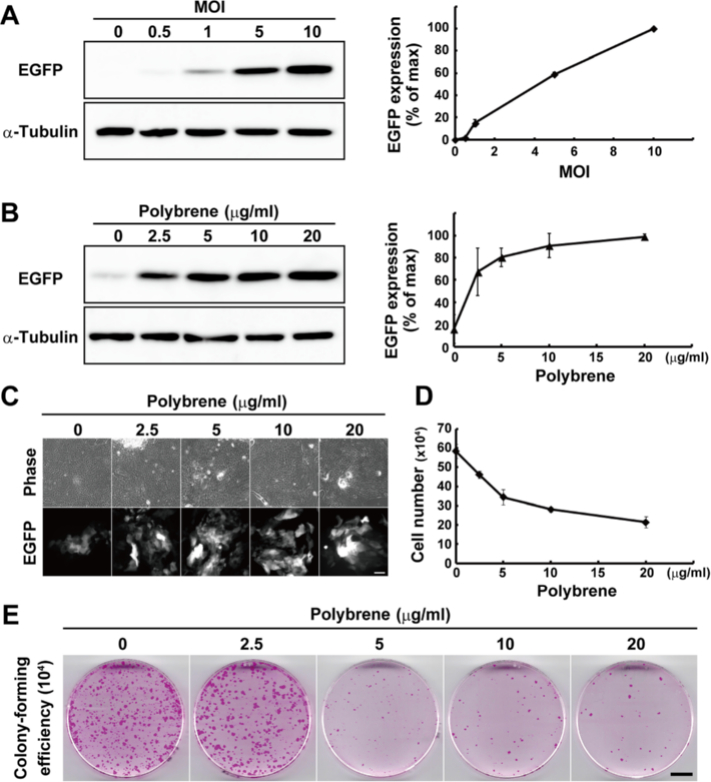
**The effects of polybrene on gene transduction efficiency and growth potential of keratinocytes. (A)** The left panel shows Western blotting of lysates from keratinocytes transduced with the enhanced green fluorescent protein (EGFP)-expressing lentivirus at various multiplicity of infection (MOI). The expression of α-tubulin was used as loading control. Right panel shows relative expression levels of EGFP. The values (mean ± SD) were obtained from the density of bands normalized with α-tubulin bands. **(B)** The left panel shows Western blotting of lysates from keratinocytes transduced with the EGFP-expressing lentivirus at MOI 1, and various concentration of polybrene. The expression of α-tubulin was used as loading control. Right panel shows relative expression levels of EGFP. The values (mean ± SD) were obtained from the density of bands normalized with α-tubulin bands. **(C)** Images of phase-contrast and EGFP expression in keratinocytes transduced with the EGFP-expressing lentivirus at MOI 1, and various concentrations of polybrene. Bar, 100 μm. **(D)** The effects of polybrene on keratinocyte proliferation are shown. Keratinocytes (10^4^ cells) were cultivated with various concentration of polybrene during lentivirus transduction, and the number of keratinocytes were counted after seven days of infection. **(E)** Determination of colony-forming efficiency (CFE) of keratinocytes infected with lentiviral particles at MOI 1, and various concentrations of polybrene. After seven days of infection, 10^4^ cells were passaged and cultured without polybrene for eight days until the cultures were fixed and stained with rhodamine B. Bar, 10 mm. The values (means ± SD) in A, B, and D were determined based on results from triplicate experiments.

To evaluate gene transduction with a lentiviral vector, we prepared a lentiviral vector expressing EGFP under the control of the cytomegalovirus (CMV) promoter. After four hours of seeding on a feeder layer of mitomycin C-treated 3 T3 cells, keratinocytes were infected with the EGFP-expressing virus at various MOI, and the expression of EGFP was analyzed by Western blotting after seven days of culture (Figure 1). Increased expression levels of EGFP correlated with the increase in the MOI (Figure 2A). Less than 10% of keratinocytes expressed EGFP at MOI 1 (Figure 2C), a condition in which almost all HEK293 cells expressed EGFP. We examined the effects of polybrene on lentiviral gene transduction at MOI 1 into human epidermal keratinocytes. Polybrene was added into the medium in which keratinocytes were incubated overnight with lentiviral particles. The next day, the medium containing polybrene and the lentiviruses was removed and replaced with fresh medium. Keratinocytes were further cultivated for six days, and the expression of EGFP was analyzed by Western blotting. As the concentration of polybrene increased, the expression levels of EGFP also increased (Figure 2B, C). An overnight treatment with polybrene for lentiviral transduction, however, inhibited the proliferation of keratinocytes, even if keratinocytes were treated with only 2.5 μg/ml of polybrene (Figure 2D). We further investigated the effect of polybrene on the growth potential of keratinocytes. Keratinocytes were treated with polybrene overnight for lentiviral transduction at MOI 1, and the same number of keratinocytes (10^4^ cells) was passaged into a new 6-cm cell culture dish after seven days of culture. The keratinocytes were then maintained without polybrene for eight days until the culture was fixed and stained with rhodamine B. The results indicated that transient treatment of keratinocytes with polybrene negatively affected the maintenance of growth potential of human keratinocytes at a concentration higher than 5 μg/ml (Figure 2E). Collectively, the treatment of keratinocytes with 2.5 μg/ml of polybrene could enhance lentiviral transduction efficiency significantly and maintain their growth potential.

**Figure 3:**
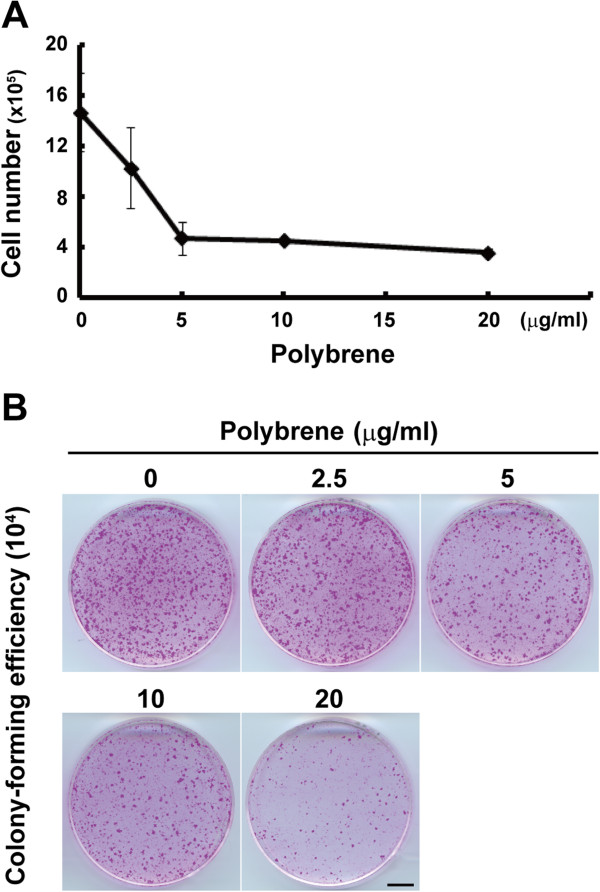
**Polybrene negatively impacts the proliferation and maintenance of growth potential of human keratinocytes. (A)** The effects of polybrene on keratinocyte proliferation are shown. Keratinocytes (2 × 10^5^ cells) were seeded and treated with various concentration of polybrene overnight, and then the number of keratinocytes were counted after seven days of cultivation. The values (means ± SD) were determined based on results in triplicate experiments. **(B)** Determination of colony-forming efficiency (CFE) of keratinocytes cultivated with various concentrations of polybrene is shown. After seven days of cultivation with the condition described above, 10^4^ cells were passaged and cultured without polybrene for eight days until the cultures were fixed and stained with rhodamine B. Bar, 10 mm.

We next examined whether negative effects of polybrene on keratinocyte proliferation and growth potential resulted from the toxicity of EGFP expression in transduced keratinocytes. An overnight treatment with polybrene after seeding markedly inhibited the proliferation of untransduced keratinocytes (Figure 3A), as shown in transduced keratinocytes (Figure 2D). Furthermore, as the concentration of polybrene increased, the colony-forming efficiency (CFE) decreased (Figure 3B). These data confirmed that polybrene itself negatively impacted the proliferation and maintenance of growth potential of human keratinocytes regardless of the EGFP expression.

### Clonal expansion of keratinocyte stem/progenitor cells expressing a transgene

**Figure 4:**
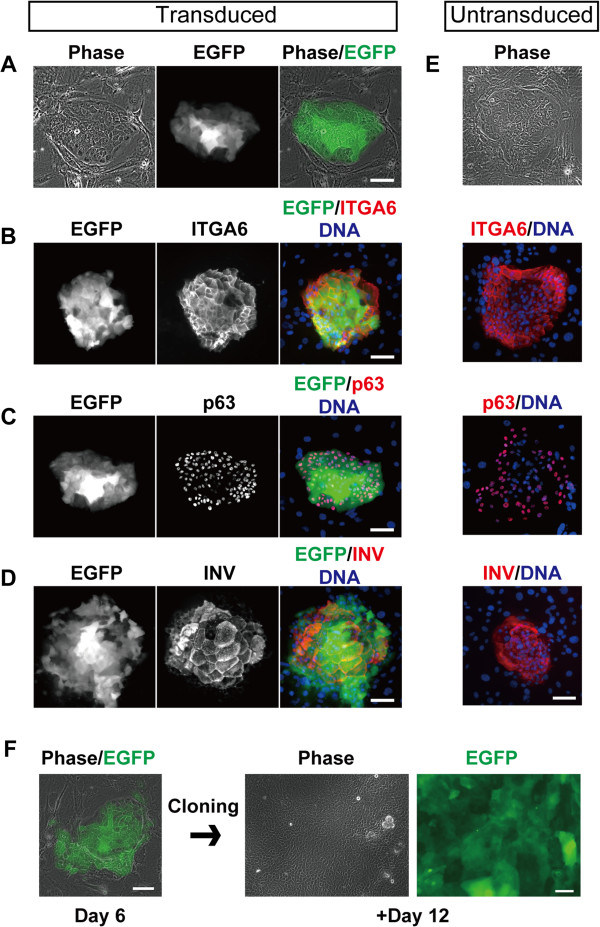
**The maintenance of self-renewing and terminal differentiation abilities in keratinocyte stem/progenitor cells expressing EGFP. (A)** Images of phase-contrast (left column) and enhanced green fluorescent protein (EGFP) expression (middle column) in a progressively growing colony. The merged image (right column) shows clonal expansion of EGFP-expressing single keratinocytes. Keratinocytes (2 × 10^3^ cells) were seeded on mitomycin C-treated 3 T3 cells, and transduced with the EGFP-expressing lentiviral vector at multiplicity of infection (MOI) 1. After six days of cultivation, the expression of EGFP and keratinocyte marker proteins were analyzed by immunofluorescence microscopy. **(B-D)** The left column shows the expression of EGFP in growing colonies. The middle column shows the expression of ITGA6 **(B)**, p63 **(C)**, or involucrin (INV) **(D)**. DNA was also visualized with Hoechst 33258, and merged images (right column) show clonal expansion of EGFP-expressing single keratinocytes. **(E)** The phase-contrast image and the expression of ITGA6, p63 and INV in progressively growing colonies derived from untransduced keratinocyte stem/progenitor cells. Bars, 100 μm. **(F)** Long-term maintenance of EGFP expression in keratinocyte stem/progenitor cells. The EGFP-expressing colony was isolated, trypsinized and expanded in a new culture dish. EGFP expression was maintained for at least 18 days after the lentivirus was transduced. Bars, 100 μm.

Keratinocytes were seeded at clonal density, and transduced with the EGFP-expressing lentivirus. In a condition in which lentiviruses were infected into keratinocytes at MOI 1 in the presence of 2.5 μg/ml of polybrene, progressively growing colonies expressed EGFP in almost all cells (Figure 4A). Keratinocytes could be identified as α6 integrin (ITGA6)-expressing cells [22] (Figure 4B). These growing colonies are formed by keratinocyte stem/progenitor cells [18]. We confirmed that EGFP-expressing growing colonies expressed p63, a transcriptional factor that is expressed in keratinocyte stem cells [23], and is essential for significant growth capacity of keratinocytes [24] (Figure 4C). EGFP-expressing growing colonies also possessed the ability to stratify and express a terminal differentiation marker, involucrin (INV) [25] (Figure 4D). The expressions of these marker proteins in the colony derived from EGFP-expressing keratinocyte stem/progenitor cells were completely identical to those in progressively growing colonies formed by untransduced keratinocyte stem/progenitor cells (Figure 4E). We further confirmed the long-term maintenance of EGFP expression in keratinocytes after cloning of an EGFP-expressing keratinocyte colony (Figure 4F). These results indicated that keratinocyte stem/progenitor cells carrying a transgene could be maintained long-term in the presence of 2.5 μg/ml of polybrene during lentiviral transduction.

### Lower transduction efficiency of 3 T3 feeder cells

**Figure 5:**
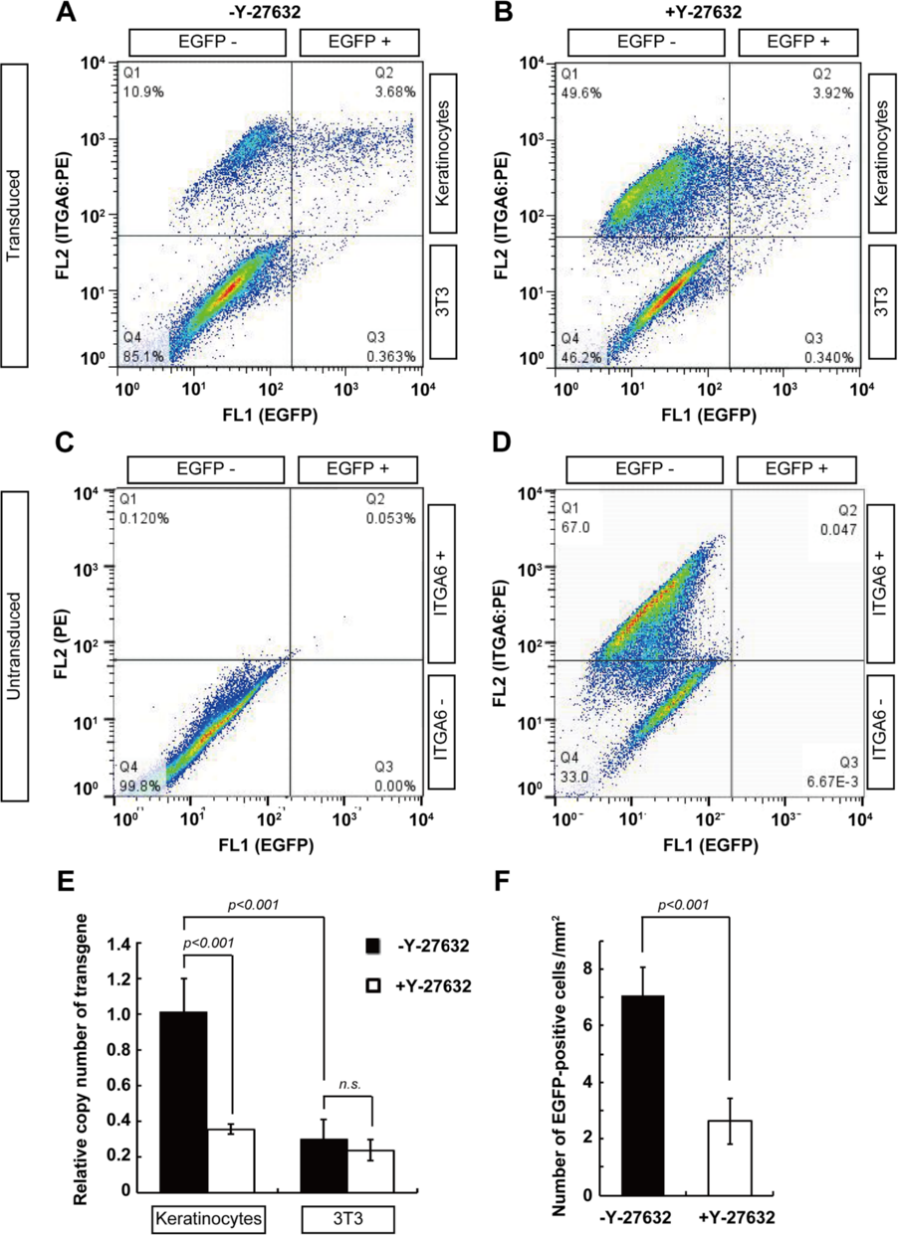
**The interference of a ROCK inhibitor with lentiviral gene transduction. (A-D)** Flow cytometric analysis of keratinocyte culture on a feeder layer of 3 T3 fibroblasts in the presence **(B)** and absence **(A)** of Rho-associated kinase (ROCK) inhibitor Y-27632. Keratinocytes (5 × 10^5^ cells) were seeded on mitomycin C-treated 3 T3 cells, and transduced with the enhanced green fluorescent protein (EGFP)-expressing lentiviral vector at multiplicity of infections (MOI) 1, with and without 10 μM of Y-27632. After seven days of cultivation, the expression levels of EGFP and ITGA6 were analyzed by flow cytometry. ITGA6-positive and -negative cells were recognized as keratinocytes and 3 T3 fibroblasts, respectively. Representative dot plot images of triplicate experiments in each experimental condition are shown. Numerical values indicate the percentage of cells in each subpopulation. **(C-D)** Negative controls of flow cytometric analysis of keratinocyte culture on a feeder layer of 3 T3 fibroblasts shown in Figure 3. Keratinocytes (5 × 10^5^ cells) were seeded on mitomycin C-treated 3 T3 cells, grown for seven days, and analyzed by flow cytometry. Flow cytometric analysis of the untransduced suspended keratinocytes and 3 T3 fibroblasts incubated with only PE-conjugated secondary antibodies **(C)**, or with PE-conjugated secondary antibodies after treatment with anti-ITGA6 antibodies **(D)**. Numerical values indicate the percentage of cells in each subpopulation. **(E)** Quantitative PCR analysis of relative copy number of EGFP transgene in keratinocytes and 3 T3 fibroblasts in the presence (□) and absence (■) of 10 μM of Y-27632. **(F)** The number of EGFP-positive cells in the keratinocyte cultures after two days of lentiviral transduction in the presence and absence of 10 μM of Y-27632. *P-*value was calculated by Student’s *t*-test. *n.s.*, not significant. The values (means ± SD) in E and F were determined based on results from triplicate experiments.

Lentiviral transduction enables a transgene to permanently integrate into the genome of cells, even if the cells do not proliferate [26]. However, the expression of EGFP was mainly observed in keratinocyte colonies, but not in the feeder cells, after seven days of transduction as shown above (Figure 4A-D). The lower transduction efficiency of 3 T3 feeder cells favors the generation and transplantation of genetically engineered keratinocyte stem/progenitor cells because transduction of a gene that functions in keratinocytes into the 3 T3 feeder cells might cause unexpected effects. To confirm the difference in transduction efficiency between keratinocytes and mitomycin C-treated 3 T3 fibroblasts precisely, we evaluated the percentage of EGFP-expressing cells in both cell types by flow cytometry. Keratinocytes were recognized as ITGA6-positive cells in a mixed cell population containing keratinocytes and fibroblasts [27]. In a condition in which lentiviruses were infected into keratinocytes at MOI 1 in the presence of 2.5 μg/ml polybrene, 23.4 ± 2.62 (mean ± SD)% of ITGA6-positive keratinocytes expressed EGFP (Figure 5A and See Methods). However, the expression of EGFP was only detectable in 1.64 ± 1.71 (mean ± SD)% of ITGA6-negative 3 T3 fibroblasts (Figure 5A and See Methods).

### ROCK inhibitor interferes with lentiviral transduction into keratinocytes

The addition of the ROCK inhibitor Y-27632 into the culture medium results in the increased proliferation of human keratinocytes [28-30]. The mechanism is still unknown; however, it could have investigational and medical applications. Therefore, we examined the effect of Y-27632 on lentiviral transduction into human keratinocytes. Lentiviruses encoding EGFP cDNA were infected with or without 10 μM Y-27632, and the efficiency of gene transduction was analyzed by flow cytometry after seven days of cultivation (Figure 5A-D). In a condition in which lentiviruses were infected into keratinocytes at MOI 1 in the presence of 2.5 μg/ml polybrene, 23.4 ± 2.62% (mean ± SD) of ITGA6-positive keratinocytes expressed EGFP (Figure 5A and See Methods). However, only 8.51 ± 1.68% (mean ± SD) of ITGA6-positive keratinocytes cultured in the presence of 10 μM Y-27632 expressed EGFP (Figure 5B and See Methods). We further examined the integration of the transgene gene into the genome of keratinocytes and 3 T3 fibroblasts, with or without Y-27632. Quantitative PCR revealed that the integration of the exogenous EGFP gene into keratinocytes was significantly interfered with the treatment of Y-27632 (Figure 5E). This analysis also confirmed that EGFP-expressing lentiviruses easily infected into keratinocytes, compared to 3 T3 fibroblasts (Figure 5A), as shown in Figure 3. Interestingly, Y-27632 inhibited lentiviral transduction into keratinocytes, but not into a feeder layer of 3 T3 fibroblasts (Figure 5B, E). We also counted the number of EGFP-positive cells in the keratinocyte cultures two days after lentiviral transduction with or without 10 μM Y-27632, and confirmed that Y-27632 inhibited lentiviral transduction (Figure 5F). This result indicated that the decreased ratio of EGFP-expressing keratinocytes with Y-27632 after seven days of cultivation was not mainly due to the increased expansion of untransduced keratinocytes with Y-27632.

### A combination of polybrene and Y-27632 efficiently expands keratinocytes carrying a transgene

**Figure 6:**
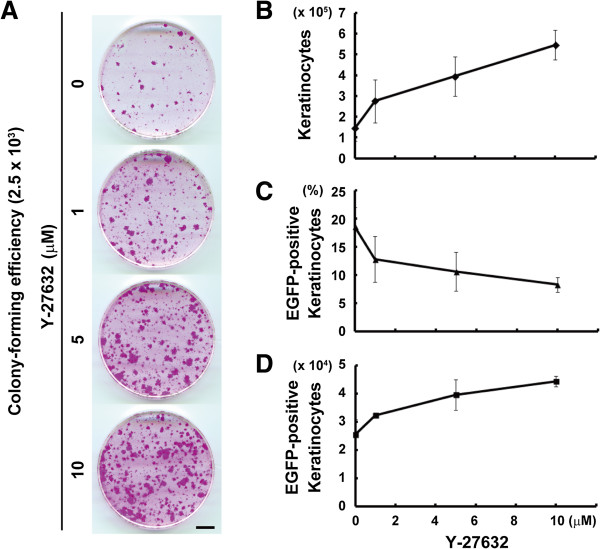
**Efficient expansion of keratinocytes carrying EGFP with a combination of polybrene and Y-27632. (A)** Determination of colony-forming efficiency of keratinocytes treated with various concentrations of Y-27632 for four days, after the first day of cultivation. A total of 2.5 × 10^3^ keratinocytes were seeded and cultured for 12 days until the cultures were fixed and stained with rhodamine B. Bar, 10 mm. **(B-D)** Flow cytometric analysis of keratinocytes cultured on a feeder layer of 3 T3 fibroblasts with various concentrations of Y-27632. Keratinocytes (5 × 10^5^ cells) were cultivated with various concentrations of Y-27632 during lentivirus transduction at multiplicity of infections (MOI) 1, and then the keratinocyte cultures were analyzed after seven days of transduction. ITGA6-positive and -negative cells were recognized as keratinocytes and 3 T3 fibroblasts, respectively. The quantitative representation of the effects of Y-27632 on keratinocyte proliferation **(B)**, transduction efficiency into the keratinocytes **(C)** and the expansion of enhanced green fluorescent protein (EGFP)-expressing keratinocytes **(D)**. The values (means ± SD) were determined based on results from triplicate experiments.

We confirmed that Y-27632 increased colony-forming efficiency and keratinocyte proliferation, even if used at 1.0 μM (Figure 6A). Finally, we explored the condition in which keratinocytes carrying EGFP transgene were expanded the most efficiently. EGFP-expressing lentiviruses were infected into keratinocytes at MOI 1 in the presence of 2.5 μg/ml polybrene and various concentrations of Y-27632. Keratinocytes were treated with Y-27632 for four days after the first day of cultivation. We evaluated the efficiency of gene transduction into keratinocytes by flow cytometry after seven days of cultivation. Although the number of keratinocytes increased (Figure 6B), the percentage of EGFP-positive keratinocytes decreased as the concentration of Y-27632 increased (Figure 6C and See Methods). However, the net number of EGFP-expressing keratinocytes increased as the concentration of Y-27632 increased (Figure 6D), and still did not reach a plateau by treatment with 20 μM of Y-27632 (Figure 6D). The effects of higher concentrations of Y-27632 should be tested. These data clearly indicated that a combination of 2.5 μg/ml polybrene and Y-27632 effectively expanded keratinocytes carrying EGFP transgene.

## Discussion

Gene transduction with lentiviral vectors has already been used for basic and clinical studies of human keratinocytes. In this study, we demonstrated the efficient expansion of human keratinocyte stem/progenitor cells carrying a transgene with lentiviral vector by using polybrene and Y-27632. This work has three major findings concerning lentiviral transduction into keratinocytes. First, polybrene markedly enhances the efficiency of lentiviral gene transduction, but reduces keratinocyte proliferation and negatively impacts the maintenance of keratinocyte stem/progenitor cells at a concentration higher than 5 μg/ml. Second, ROCK inhibitor Y-27632 significantly interferes with the lentiviral transduction into cultured human keratinocytes. Third, a suitable combination of polybrene and Y-27632 can effectively expand keratinocytes carrying a transgene.

Polybrene is widely used in viral gene transduction. Polybrene has no cytotoxic activity at low concentrations, but negatively affects the proliferation of corneal keratinocytes at concentrations greater than 10 μg/ml [31]. It also negatively impacts the proliferation of human mesenchymal stem cells, even if used at 1 μg/ml [32]. It has been reported that treatment with 2 to 6 μg/ml of polybrene results in the highest efficiency of gene transduction with adenoviruses in human epidermal keratinocytes [14]. In our experiments, 20 μg/ml of polybrene was required to induce the highest expression of transgene in lentiviral transduction. However, polybrene significantly reduced the proliferation of keratinocytes, even if used at low concentrations. Furthermore, we demonstrated that polybrene negatively impacted the maintenance of the keratinocyte stem/progenitor cells at a concentration higher than 5 μg/ml. In serial culture, keratinocyte stem cells progressively lose their proliferative capacity to become transient amplifying cells with limited growth, a phenomenon termed clonal conversion [33]. Clonal conversion is accelerated by stress, suboptimal culture conditions, serial cultivation and age of donor [33]. It is also governed by the balance of Rac1 and Akt signaling activity and actin filament organization [18]. As polybrene modulates the charge of the cell surface, it might affect these intracellular signaling pathways and actin network, and then increase the rate of clonal conversion. Retroviral gene transfer into keratinocytes is also enhanced by another polycationic polymer, protamine sulfate [31] and by their cultivation on a substrate of fibronectin [16]. The effects of these reagents on transduction efficiency and clonal conversion should be further investigated.

The process of retroviral infection involves the absorption of viral particles on the host plasma membrane, the entry of the viral protein-RNA complexes into the cytoplasm, the reverse transcription of viral RNA into DNA and the intracellular trafficking and entry of viral protein-DNA complexes into the nucleus, which is followed by the integration of viral DNA into the host genome [26]. We have clearly demonstrated here that the ROCK inhibitor Y-27632 interfered with the lentiviral transduction in human epidermal keratinocytes. ROCK regulates both actin polymerization and phosphorylation of myosin regulatory light chain [34]. Recently, it has also been shown that ROCK regulates microtubule dynamics through phosphorylation of the tubulin polymerization promoting protein 1 (TPPPA/p25) [35]. The cytoskeleton, including actin filaments and microtubules in the host cells, contributes to intracellular transport of retroviral genomes from the cytoplasm into the nucleus [36-38]. As further investigations should be required, ROCK activity could be involved in intracellular trafficking of the retroviral genome.

Interestingly, van den Bogaard *et al*. have recently reported that Y-27632 enhances lentiviral transduction into human keratinocytes [17]. This report seems to be contrary to our results. However, van den Bogaard *et al*. have used high-passage adult keratinocytes for lentivirus transduction and the generation of human skin equivalents (HSEs), and then assessed the efficiency of lentiviral transduction into keratinocytes in HSEs, but not in cultured keratinocytes. Human adult keratinocytes significantly decrease their proliferative capacity by serial cultivation and stress in culture, including gene transduction [33]. Therefore, the enhanced expansion of EGFP-expressing adult keratinocytes in HSEs by Y-27632 might be due to the fact that the expansion of Y-27632-treated adult keratinocytes expressing EGFP is more efficient than that of untreated EGFP-expressing adult keratinocytes during the formation of HSEs.

van den Bogaard *et al*. has also reported high levels of GFP expression in the differentiated layers of HSEs [17]. We also observed that EGFP was strongly expressed in stratified and differentiating cells in the center of colonies. These results suggest that CMV promoter activity is enhanced in the stratified and differentiating keratinocytes. Y-27632 inhibits keratinocyte differentiation [28-30]. These observations alone suggest that Y-27632 inhibits EGFP expression under the control of the CMV promoter, but not lentiviral transduction itself. However, our quantitative PCR analysis has revealed that the integration of the exogenous EGFP gene into keratinocytes significantly interfered with the treatment of Y-27632. Consequently, reduced expression of EGFP after lentiviral transduction with Y-27632 might be, at least partially, due to the inhibition of keratinocyte differentiation and, therefore, CMV promoter activity by Y-27632, as well as lower transduction efficiency by the treatment with Y-27632.

We have demonstrated in this study that keratinocytes gave rise to colonies derived from their stem/progenitor cells carrying a transgene after lentiviral transduction with lower concentration of polybrene. We have also shown that the keratinocyte stem/progenitor cells carrying EGFP transgene could maintain both self-renewing and terminal differentiation abilities that are indispensable for making a functional epidermal sheet *ex vivo*, and permanent engraftment after transplantation [33,39]. These results indicate that a homologous clone of a keratinocyte stem cell carrying a transgene can be isolated and expanded massively for cell therapy for genetic disorders of the skin [7,40].

## Conclusions

A suitable combination of polybrene and Y-27632 efficiently expands keratinocyte stem/progenitor cells carrying a transgene, although polybrene and Y-27632 negatively affect growth and lentiviral transduction of human keratinocyte stem/progenitor cells, respectively. This point is particularly significant for the application of genetically modified keratinocyte stem/progenitor stem cells in regenerative medicine.

## Abbreviations

CFE: Colony-forming efficiency; CMV: Cytomegalovirus; EGFP: Enhanced green fluorescent protein; HBSS (+): Hank’s balanced salt solution containing calcium and magnesium; HRP: Horseradish peroxidase; HSEs: Human skin equivalents; INV: Involucrin; ITGA6: Integrin alpha 3; LAMB3: Laminin beta 3; MOI: Multiplicity of infection; PE: Phycoerythrin.

## Competing interests

The authors declare that they have no competing interests.

## Authors’ contributions

DN and NM designed the experiments. DN and FT performed cell culture experiments, Western blotting and flow cytometry. NM produced lentiviruses and performed qPCR analysis. DN and SH analyzed and interpreted the data. DN wrote the manuscript. All authors read and approved the final manuscript.
